# Understanding discarding in trawl fisheries: A model based demersal case study with implications for mitigating and assessing impacts

**DOI:** 10.1371/journal.pone.0264055

**Published:** 2022-02-17

**Authors:** Thomas C. Barnes, Steven G. Candy, Stephen Morris, Daniel D. Johnson

**Affiliations:** 1 New South Wales Department of Primary Industries, Port Stephens Fisheries Institute, Nelson Bay, NSW, Australia; 2 Institute of Marine and Antarctic Studies, University of Tasmania, Hobart, Tasmania, Australia; 3 SCANDY STATISTICAL MODELLING PTY LTD, Blackmans Bay, Tasmania, Australia; COISPA Tecnologia & Ricerca - Stazione Sperimentale per lo Studio delle Risorse del Mare, ITALY

## Abstract

Despite research and public scrutiny over recent decades, discarding continues to be an issue for trawl fisheries. Previous research demonstrates that environmental, biological, operational, legislative and socioeconomic drivers affect a fisher’s decision to discard an organism. Therefore, the reduction of fishery discards requires a better understanding of fishery-specific drivers. Despite considerable research and mitigation, further work is required to reduce discarding to acceptable levels (currently ~ 50% in Australia). To better understand the drivers of discarding, this study used a modelling approach to determine environmental and operational factors that drive discarding in the New South Wales (NSW) ocean prawn trawl fishery (OPT). Further, the study investigated the relationship between the discarded number of individuals from all functional species groups (i.e. elasmobranchs, crustaceans and fish combined) and the retained catch weight. This model was also run on just fish partly due to their disproportionally high contribution to the discard assemblage (e.g. 76% of all species or higher taxon) and importance (e.g. to the ecosystem and fisheries). The results quantified relationships of environmental and operational drivers of discarding and the relationship of fish discarding and retained catch weight was found to be linear. However, the identified relationships appear complicated and, whilst an important first step, more work is required to identify all drivers influencing discarding practices. We, in combination with previous research, suggest implementation of effort quotas may be a suitable management initiative to reduce discarding and its impact; at least whilst more research is conducted to better understand this complex process. Furthering our understanding of discarding is urgent given its global impact and the rate of discarding in the OPT.

## Introduction

The discarded portion of penaeid and other-trawl catches consist of non-marketable organisms. Discards include non-commercial species and commercial species that are regulated by size or reproductive stage (e.g. ovigerous female crustaceans), or may have been damaged during trawling [1,2]. Discarding has been occurring since fishing began [3]. The decision to discard or retain a particular species relies on a complex combination of driving factors (e.g. biological, legislative, and socioeconomic) [4,5]. Further, variations in discarding have been shown to be a result of environmental fluctuations and operational (vessel configuration and skipper decision making) differences [e.g. 6]. Tropical and sub-tropical penaeid trawls are known to produce a high proportion of discards due to the diverse array of species that cohabit with penaeids [2,3,7,8]. Discarding is seen as a wasted resource and can have negative impacts on the marine ecosystem (e.g. changes to trophic structure and habitats): hence, there has been global attention on the penaeid-trawl industry (amongst other demersal trawl fisheries) [2,4] for the last three decades [9,10]. In this study, the term discarding refers to the practise of returning an organism to the sea after capture.

A key issue with discarding is the associated mortality of many organisms [2,11,12]. Discard survival is low, especially for teleost fish (herein fish) and some crustaceans [13–15]. However, the survival of some discarded species groups is high; for example, previous research has shown that the survival of some elasmobranchs is high [16–18], including ~ 90% survival of discarded eastern shovelnose rays [19].

The low survivorship of organisms from discarding is likely to have a negative effect on the ecosystem [1,20]. Therefore, it is important to manage discarding to avoid potential large-scale threats to marine biodiversity. Specifically, poorly managed discarding has negatively affected other fisheries [2]. For example, specimens below minimum legal size (MLS) snapper (*Chrysophrys auratus*) a species that support large fisheries in Australasia, have been reported to have a high mortality rate (~85%) when discarded in an Australian penaeid trawl fishery [21]. Mortality of fishery species from discarding potentially represents unquantified impacts on commercial and recreational stocks. Such mortality data should be included in stock assessments to accurately quantify fishing mortality. However, this is not always the case [22] and measurements of discarding from targeted monitoring programmes are often not available [10].

The United Nations Food and Agriculture Organisation’s (FAO) most recent estimation of Australian discarding was ~ 55% of the total catch [7] and more recent estimated proportions are still high (> 40%) [10], despite adoption of bycatch reduction devices (BRDs) in some fisheries. Global discard proportions have been quoted at ~ 10% but data was from a small proportion of fisheries [23,24]. There is scope to further reduce discarding which may be facilitated by a better understanding of the complexities of discard drivers [e.g. 25]. Not surprisingly, the necessity to reduce discarding, has been recognised at the international level such as the FAOs International Guidelines on Bycatch Management and Reduction of Discards [26] and urgent action is required in demersal trawl fisheries [23]. The European Union (EU) has recently taken an aggressive approach to reduce trawl and other fishery discarding [27,28]. The EU recognises the potential economic and ecosystem impact of discarding and has implemented a discard ban (or landing obligation) where some species must be retained by the fishers (e.g. commercial species subjected to quota or MLS) [27]. Also, Marine Stewardship and eco-labelling rate the sustainability of some seafood which is increasingly assessed by the consumer community [1,10,29].

A combination of physical gear modifications and management strategies have been used to reduce discards. Bycatch reduction devices, including turtle excluder devices (TEDs) [e.g. 30], are mandated in many penaeid-trawl fisheries that allow a portion of the discarded catch to escape [9,31]. Spatial management measures have also been implemented to reduce discarding due to the spatiotemporal variation in the abundance of many marine organisms. For example, Marine Protected Areas (MPAs) provide protection from trawling which results in flow-on benefits to fisheries [1,32]. Real-time spatial management tools provide a flexible alternative to more static spatial management. This approach has recently been reported to have reduced discarding in multiple fisheries by creating a variety of incentives to avoid unwanted catch [33].

As discarding occurs at sea there are limited opportunities to monitor and gather reliable data [34]. One approach is to have trained observers record specific information about discarding, including species identification and quantity [10]. Observers are increasingly recording operational and environmental factors [e.g. 6] which provides information that improves the understanding of potential drivers that could reduce discarding.

As the volume of discarding by a fishery is difficult to precisely measure, it is often estimated as a function of retained catch [10]. For example, a discarded to retained catch ratio may be estimated from a representative monitoring program which is then extrapolated to the fishery level [e.g. 7]. Mediterranean demersal trawl fisheries have comprehensive representative discard monitoring under the EU data collection framework [35] and utilise the retained catch and discarding ratio for fishery wide estimates. The extrapolation requires that a representative relationship between retained catch and discarding exists; however, this is often shown to be not the case or, concerningly, not assessed [10].

**Fig 1:**
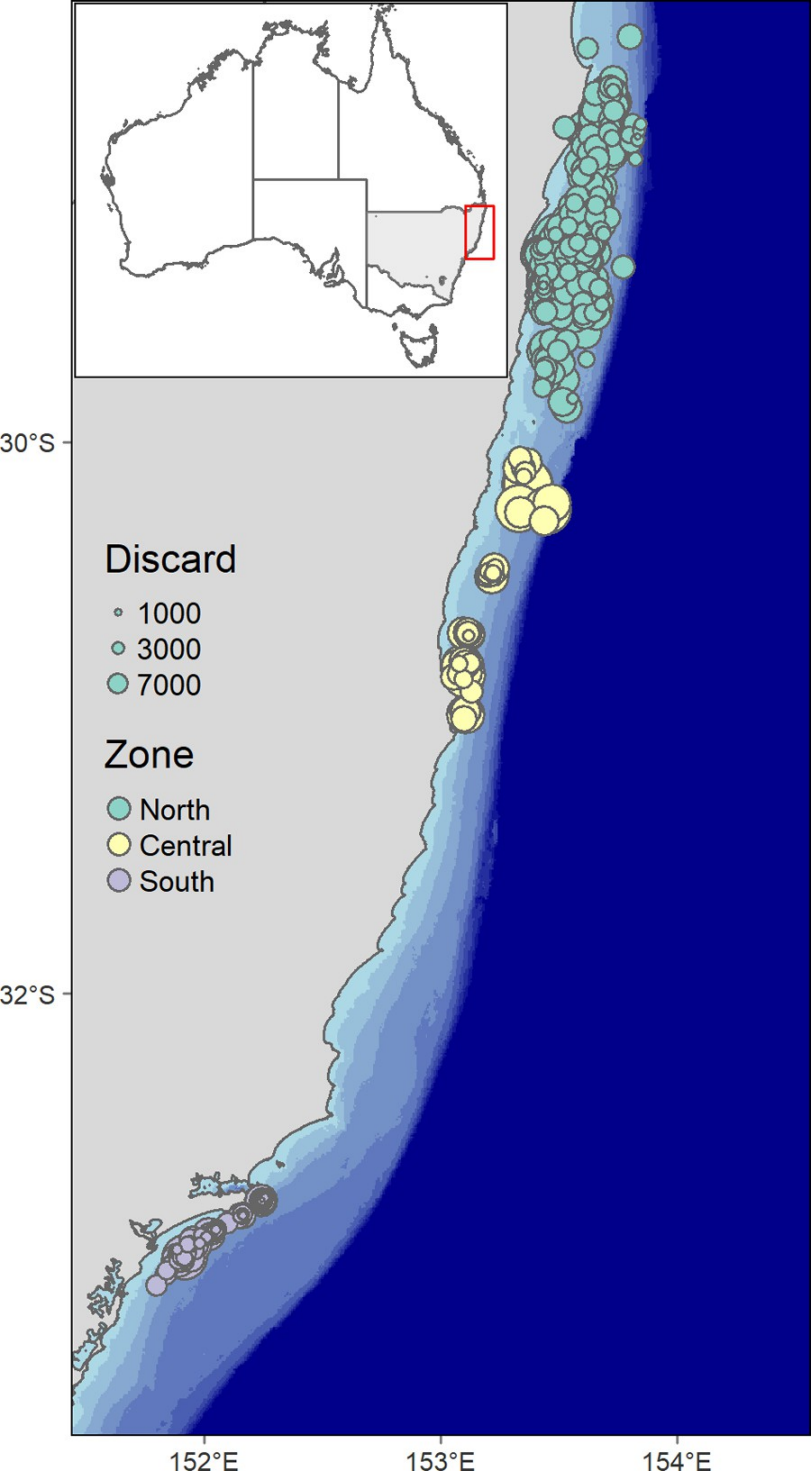
Study location showing the standardised discard rate (number of individuals standardised by swept area (see methods)) of all study species groups (elasmobranchs, crustaceans and fish combined) per trawl trip in all zones. Also shown are shaded depth increments (30 m). New South Wales is shown as grey in the inset. Shape and raster files used to create this image are available at https://data.gov.au/dataset/ds-dga-a1b278b1-59ef-4dea-8468-50eb09967f18/details and https://www.gebco.net/data_and_products/gridded_bathymetry_data/.

In New South Wales (NSW), the ocean prawn trawl fishery (OPT) is typical of global penaeid fisheries (i.e. nearshore, warm water trawl grounds, high discard rate, etc., Fig 1). A recent study indicated 66.7% of the catch generated by the OPT is discarded and that the OPT is responsible for 3.9% of the discards generated in all Australian fisheries [10]. To address this issue, fishing effort was restricted and catch limits were introduced for some bycatch (i.e. non-penaeid but commonly marketable or commercial) species in 2019. Input controls consist of effort, fleet and gear restrictions [36]. For example, trawlers must be < 20 m in length to operate in the OPT, trawl net mesh must be ≥ 40 mm and ≤ 60 mm, cod end mesh size ≤ 50 mm and have a BRD in the form of a square mesh panel, and headline length is restricted to ≤ 33 m [36]. Trawl operations mainly occur at night targeting eastern king prawns (EKP, *Melicertus plebejus*) [36]. The catch is sold and consumed predominately by the domestic market and is valued at A$ 20 m per annum. NSW accounts for approximately 17% of the total catch and 25% of effort targeting EKP in eastern Australian waters (NSW and Queensland (QLD)) [37]. Following the initial assessment of fleet-wide discarding in the OPT fishery, several BRDs were legislated for use, however, no assessment of fishery-level discarding has used contemporary observer collected data since 1994 [38]. In a recent review of threats and risks to the NSW marine environment, the OPT was prioritised as a high order risk to fish assemblages from discarding practices [39], partly due to the long lead time since the last monitoring work. The OPT was also prioritised as a medium order risk to seabirds but research has since found the risk rating to be likely excessive [40].

### Objectives

Understanding environmental and operational factors that drive discarding could aid in its reduction via informed management. To facilitate, we investigated the following hypothesis:

1) Environmental (spatial, temporal and habitat (depth)) and operational (retained catch weight, trawl speed, track complexity and engine capacity) drivers do not affect discard numbers.

To explore the relationship between the volume of retained total catch and (1) discarding of fish, and (2) discarding of the study species groups combined (elasmobranchs, crustaceans and fish), we tested the following hypothesis:

2) Retained catch and discard rate of fish, and all species groups combined are not correlated.

## Methods

### The study area

The NSW OPT primarily operates in the northern half of the state between 29 and 33°S (Fig 1). The northern extent of the fishery is bound by the NSW/QLD state border. There is substantial overlap of commercial species between NSW and QLD (for more information on the QLD fishery see [34]). For this study, the OPT is divided into three zones along ~ 1 000 km of coast, herein the northern, central and southern zones (Fig 1). There is one spatially (geographically) different output restriction; in the southern zone all teleost taxa with an MLS must be discarded. The north and central zones are only separated for the present study and catch reporting. OPT vessels can operate anywhere within the latitudinal boundary offshore to the 4 000 m depth isobath (Fig 1). However, there are a variety of temporal and spatial closures, some being in the form of MPAs and juvenile EKP closures [41]. All vessels participating in the OPT generally use triple rigged trawl gear.

### Data collection and description

The NSW Department of Primary Industries Animal Research Authority, Animal Care and Ethics Committee reference 07/03 specifically approved this study.

Trained scientific observers were assigned OPT trips between 2017 and 2019 (Table 1). To ensure sampling effort was representative of fishing effort, a theoretical, two-factor matrix was generated, allocating the number of available observed fishing trips among the zone/season combinations (see Table 1), according to a weighting relative to the differences in fishing effort. Where possible, discarded individuals were identified to species, enumerated and weighed. When large catches were landed, 30 individuals from each species were sampled and weighed. Total catch (counts and weights) for each species was extrapolated based on the weight of the sub-sample and the total weight of the catch [42]. Observers recorded environmental factors including location (decimal degrees), depth (meters, m) and time, and operational variables including track complexity (mean number of direction changes per trawl), trawl speed (knots, kts), engine capacity (kilowatts, kW) and retained catch (kilograms, kg).

**Table 1:** Number of observed ocean prawn trawl trips by fishery year, season, zone and depth (< = 75 m is shallow and > 75 m is deep).

		Shallow				Deep					
Zone	Year	Sum.	Aut.	Win.	Spr.	Sum.	Aut.	Win.	Spr.	Total trips	No. of boats
North	2017	10	0	0	14	0	0	0	0	24	9
	2018	21	41	13	27	2	5	33	5	147	14
	2019	18	42	27	1	0	10	18	0	116	8
Central	2017	0	0	0	3	0	0	0	0	3	3
	2018	8	9	1	1	0	2	3	0	24	7
	2019	4	14	15	0	0	2	0	0	35	4
South	2017	1	0	0	7	0	0	0	0	8	3
	2018	6	8	5	5	1	5	6	2	38	5
	2019	1	6	10	0	3	7	0	0	27	4
	Total									422	

Note: Sum = Summer, Aut = Autumn, Win = Winter, Spr = Spring, No. = number.

### Data analysis

#### General

All analyses were performed in R version 3.6.0 [43]. Clean data were then explored visually following the protocol outlined by Zuur et al. [44]. For example, all data (response and factors) were checked for correlation using conditional boxplots (categorical versus continuous) and a combination of pairs plots and Pearson’s correlation coefficient (continuous). All data were grouped to the trip level which removes autocorrelation potential that would violate assumptions of some statistical procedures (i.e. haul level information may lack independence as variables may depend on the previous haul). Sensitivity analysis of the effect of grouping to the trip level was assessed for depth with ~ 99% of trips trawling a very narrow depth range (S1 Fig). This was expected as vessels in this fishery are known to generally target relatively small spatial areas (fishing grounds) in any given trip (perhaps due to their small size etc.). Species were grouped into major taxa (herein species groups) for analysis: 1) all elasmobranchs (sharks and rays) combined, 2) crustaceans, 3) teleost fish, 4) commercial crustaceans and 5) commercial teleost fish. Commercial crustaceans and fish were any species that were retained for sale by the trawl business during sampling. In all models, the response variable (discarded catch) was based on counts (i.e. numbers) of individual organisms.

Catch ratios were estimated (kg) for discarded to retained catch (all target EKP and other commercial species) and for all bycatch (all species except EKP) to EKP catch. These ratios were reported in the previous OPT bycatch discarding study [38] and thus allow a direct comparison.

#### Hypothesis 1

To test the null hypothesis of no relationship between environmental and operational factors (the potential discard drivers) and discarding response variables generalised additive mixed models (GAMMs) were used via the ‘mgcv’ R package [45]. The full model took the following form:

$$\begin{array}{l} Discarding\;per\;trip\;(separately\;on\;species\;groups)\\ \;=\beta +s(track\;complexity)+s(longitude,latitude)+s(depth,season)\\ \;+s(vessel\;velocity)+s(engine\;capacity)+s(weight\;of\;catch\;retained)\\ \;+season+year+random\;effect(vessel)+offset(log(swept\;area))\\ \;+\varepsilon \end{array}$$

β is an overall intercept, s represents smooths (either cubic regression or Gaussian process for spatial kriging) as exploratory analysis suggested nonlinear relationships and ε an error term. Where appropriate, continuous factors were weighted means (trip level, e.g. depth). The swept area was modelled as an offset to standardise the response variable [34,46]. The swept area was calculated by linear distance trawled multiplied by 80% of the combined net headline length (functional net opening) [47]. Trawl discard data often displays residual overdispersion [6] hence, the negative binomial family and log link, with estimated theta were employed [48]. The full model was reduced by using the P values of factors in a backwards stepwise approach (i.e. the factors with the greatest P value were removed first). Vessel name was included in the model as a random effect. For most numeric factors the number of knots (or degrees of freedom) was set to four to create less complex results, although spatial factors had 100 knots to facilitate kriging and in one instance a numerical factor (latitude in the commercial fish model) was fitted as a linear term. This was based on exploratory analysis including a pilot model run and review of estimated degrees of freedom. The model was run on the species groups separately as the survivorship and contribution to discarding is separate for these groups [13,14].

#### Hypothesis 2

Fish and all species groups combined discarding were modelled against mean retained catch weight per trawl trip as an indicator of the robustness of extrapolating the volume of discarding from fisher reported catches. Fish were analysed separately in this exploratory model as they were the most abundant, are important (i.e. exploited by other fisheries) and data exploration suggested a positive linear relationship may exist. The modelling process was the same as that used in hypothesis 1 except the models only included the variables mentioned above and the offset was applied by dividing the response variable by the log swept area to facilitate using a negative binomial generalised linear mixed model (base R) that doesn’t have the offset function. The weight of catch retained was fitted as a linear factor (as opposed to a smooth in hypothesis 1).


$$\begin{array}{c} Discarding\;per\;trip\;(separately\;on\;fish\;and\;combined\;species\;groups)/log(swept\;area)\\ =\beta +weight\;of\;catch\;retained+random\;effect(vessel)+\varepsilon \end{array}$$

## Results

### Observer effort, environmental and operational data

Observers were present on 422 overnight OPT trips for 1 266 hauls (~ 3 hauls per trip, Table 1). There was a negative north to south gradient in the number of trips observed reflecting the amount of fleetwide OPT effort (Fig 1). Half of the observed trips occurred during 2018 with slightly less in 2019 (42%) and a comparatively small amount in 2017 (8%). Trips occurred in all austral seasons with most done in autumn (36%), then winter (31%), summer (18%) and spring (15%). Most trips were observed in shallow depths (75%) compared to deep (25%, Table 1). Thirty-nine vessels volunteered deck space to facilitate observers out of ~ 67 vessels that operated in the OPT during the monitoring period (source; NSW Department of Primary Industries Fisheries commercial catch and effort data). Across the vessels that facilitated observers, mean (± SD) operational data included, an engine capacity of 189.34 (± 57.09) kW, a vessel length of 15.36 (± 1.99) m, a trawl distance of 12 369.8 (± 4 079.43) m, a trawl depth of 64.55 (± 21.89) m, a trawl speed of 2.56 (± 1.67) kts, and a track complexity of 1.93 (± 0.55) direction changes.

### Discarded catches

Two hundred and thirty-seven taxa, which formed the five species groups (elasmobranchs, fish, crustaceans, commercial fish and commercial crustaceans), were discarded at some stage during the 23 month program (September 2017 to November 2019). There was a negative north to south and shallow to deep gradient in the number of taxa discarded (Table 2), however, crustacean discarding was slightly different. Crustaceans were discarded in greater numbers in the south compared to the central zone and were only discarded slightly more in the shallow compared to deep areas (Table 2). Eighty-six percent of all discarded taxa were in the northern zone, and 70 and 68% in the central and southern zones, respectively, whereas 95% of all taxa were discarded in the shallow zone compared to 82% in the deep zone. The number of organisms discarded for each observed trip varied between zones with some large discarding events occurring in the central and at the deeper extremity of trawling (Fig 1). Most taxa were from the fish species group (76%, Table 2). The discarded to retained (all commercial species including EKP) catch ratio was estimated at 2.1:1 (kg) whilst the overall bycatch (all species except EKP) to EKP catch ratio was 5.8:1 (kg).

**Table 2:** Number of discard species or higher taxa summarised by study groups by fishery zone and depth (< = 75 m is shallow and > 75 m is deep).

	Zone			Depth		
Functional group	North	Central	South	Shallow	Deep	Overall
Elasmobranchs	23	22	15	26	18	27
Crustaceans	28	21	24	29	27	30
Fish	154	123	121	169	150	180
Total	205	166	160	224	195	237
Commercial crustaceans	18	17	15	18	16	18
Commercial fish	71	62	60	71	77	78

### Hypothesis 1

Environmental and operational factors were significant drivers of discarding in all species group response models (Table 3). Therefore, the null hypothesis of no effect of environmental and operational drivers was rejected. Environmental drivers, latitude, and season (either as a main effect or interactive with depth or both), were drivers of discarding for all species groups. One operational driver (the retained catch) featured in the discarding models of all species groups except elasmobranchs (Table 3). The model deviance explained was low for fish (i.e. ~ 40%) and elasmobranchs, and high for commercial fish and crustaceans and commercial crustacean discarding models (~ 70%) (Table 3). Visual inspection of model diagnostic plots did not show concerning patterns or outliers. For example, the relationship between residuals and linear factors lacked fan or other concerning shape ensuring the negative binomial distribution captured the overdispersion correctly (S2 Fig).

**Table 3:** Generalised additive mixed model results for species groups relationship with environmental and operational factors (the discard drivers), shown are final reduced models (i.e. only statistically significant factors).

Response	Factors	Deviance explained
Elasmobranchs	Track complexity, longitude:latitude, season, year	46.6%
Crustaceans	longitude:latitude, depth:summer, autumn, retained catch, season	76.5%
Fish	Latitude, depth:summer, autumn, winter, spring, retained catch, season	42.2%
Commercial crustaceans	longitude:latitude, depth:summer, autumn, spring, engine capacity, retained catch, year	68.5%
Commercial fish	Track complexity, longitude:latitude, depth:winter, spring, speed, retained catch, season	64.2%

#### Environmental driver effects

**Fig 2:**
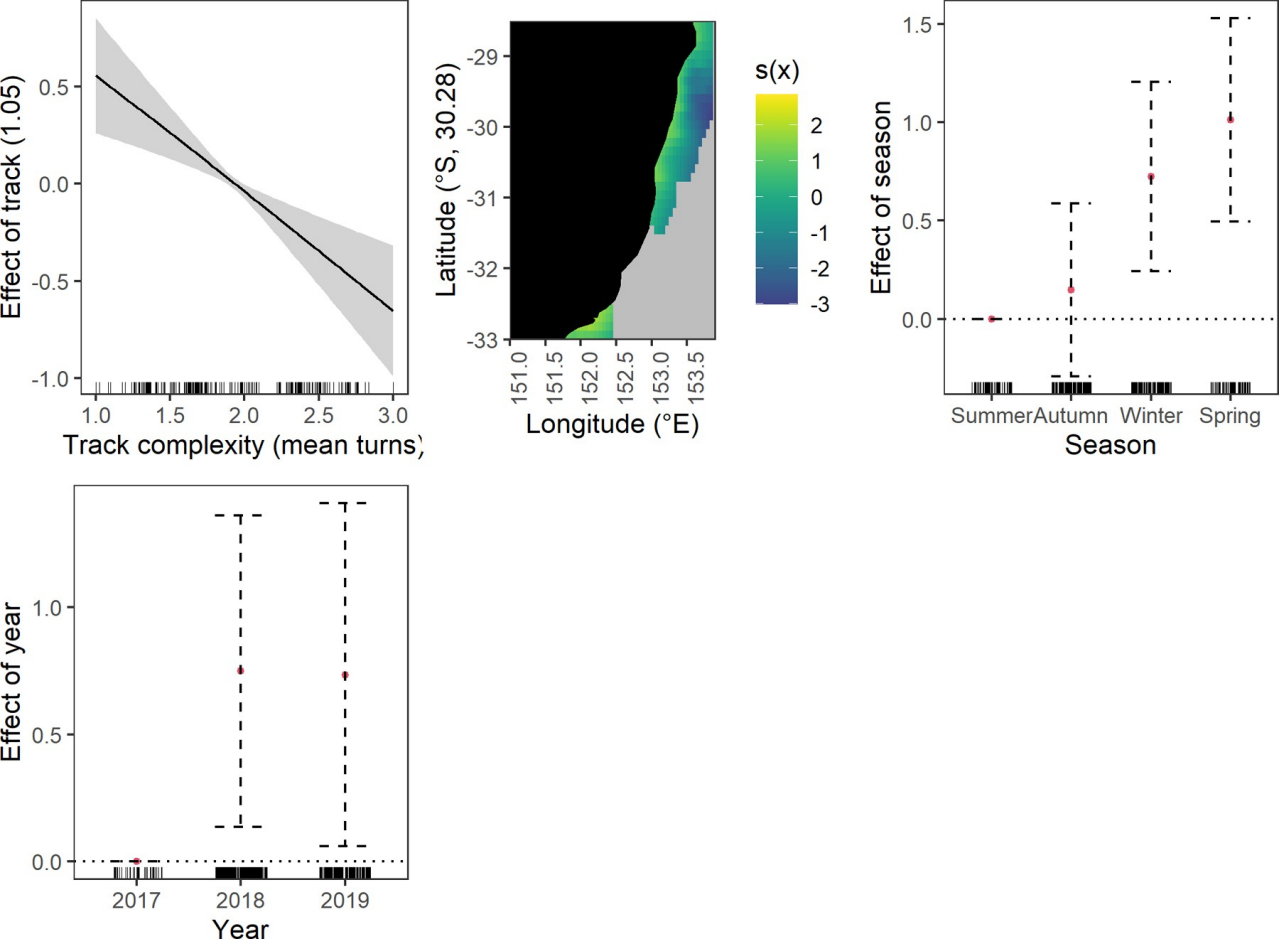
Significant effects plots from the discarded elasmobranchs response generalised additive mixed models, shaded area represents the 95% confidence limits whilst the surfaces represent the effect of 2-d smoothing on spatial coordinates.

**Fig 3:**
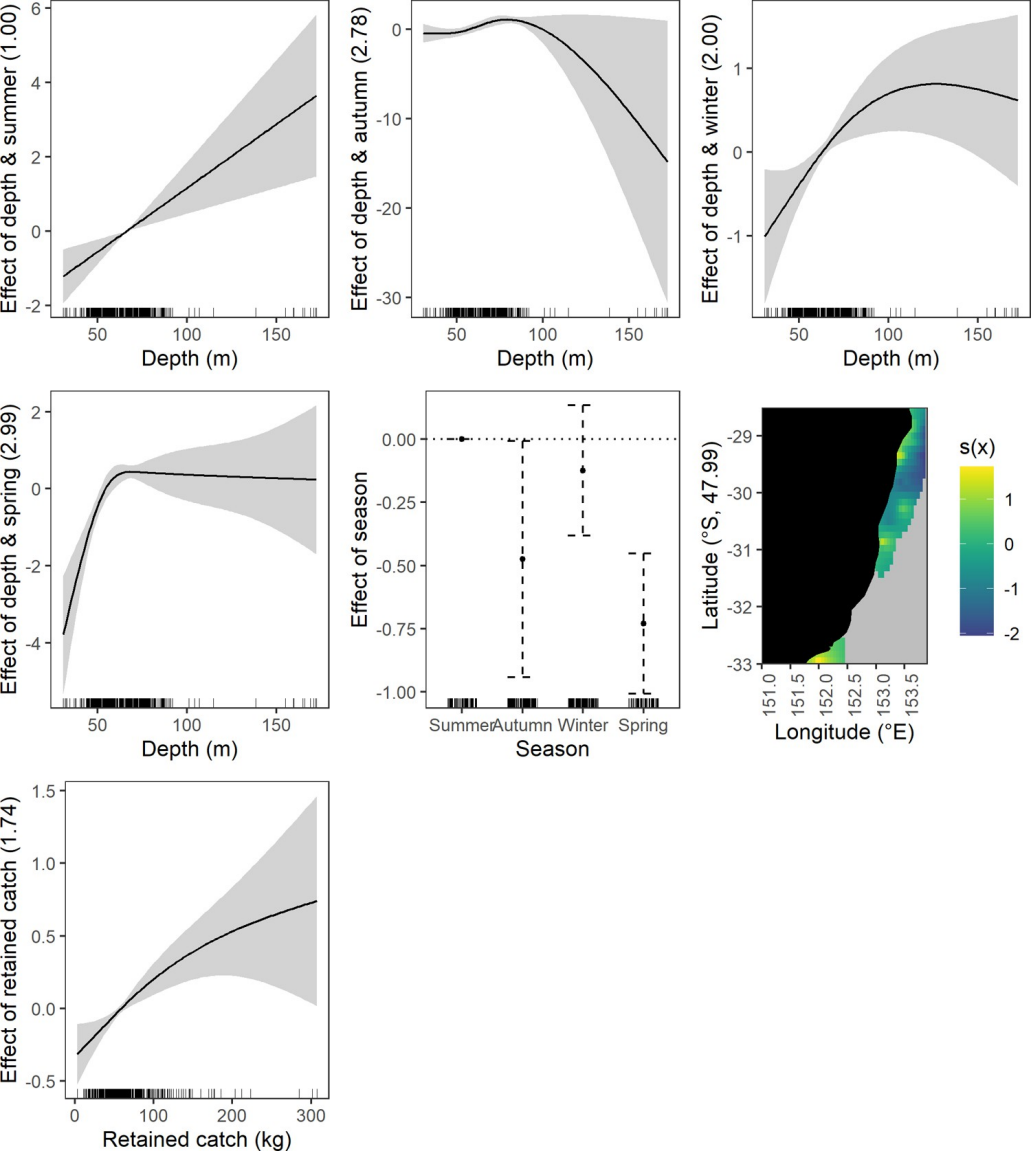
Significant effects plots from the discarded crustacean response generalised additive mixed models, shaded area represents the 95% confidence limits whilst the surfaces represent the effect of 2-d smoothing on spatial coordinates.

**Fig 4:**
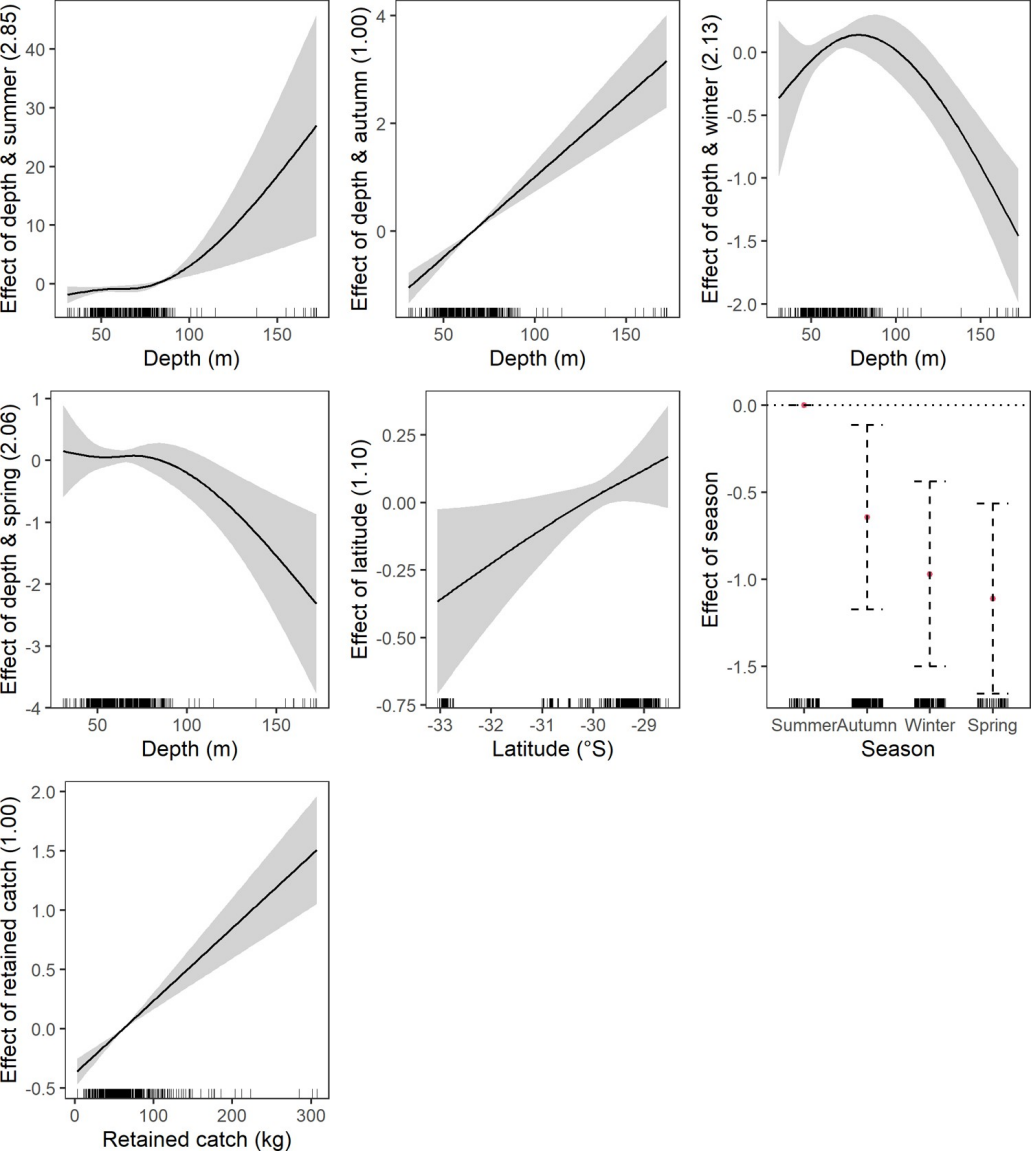
Significant effects plots from the discarded fish response generalised additive mixed models, shaded area represents the 95% confidence limits.

**Fig 5:**
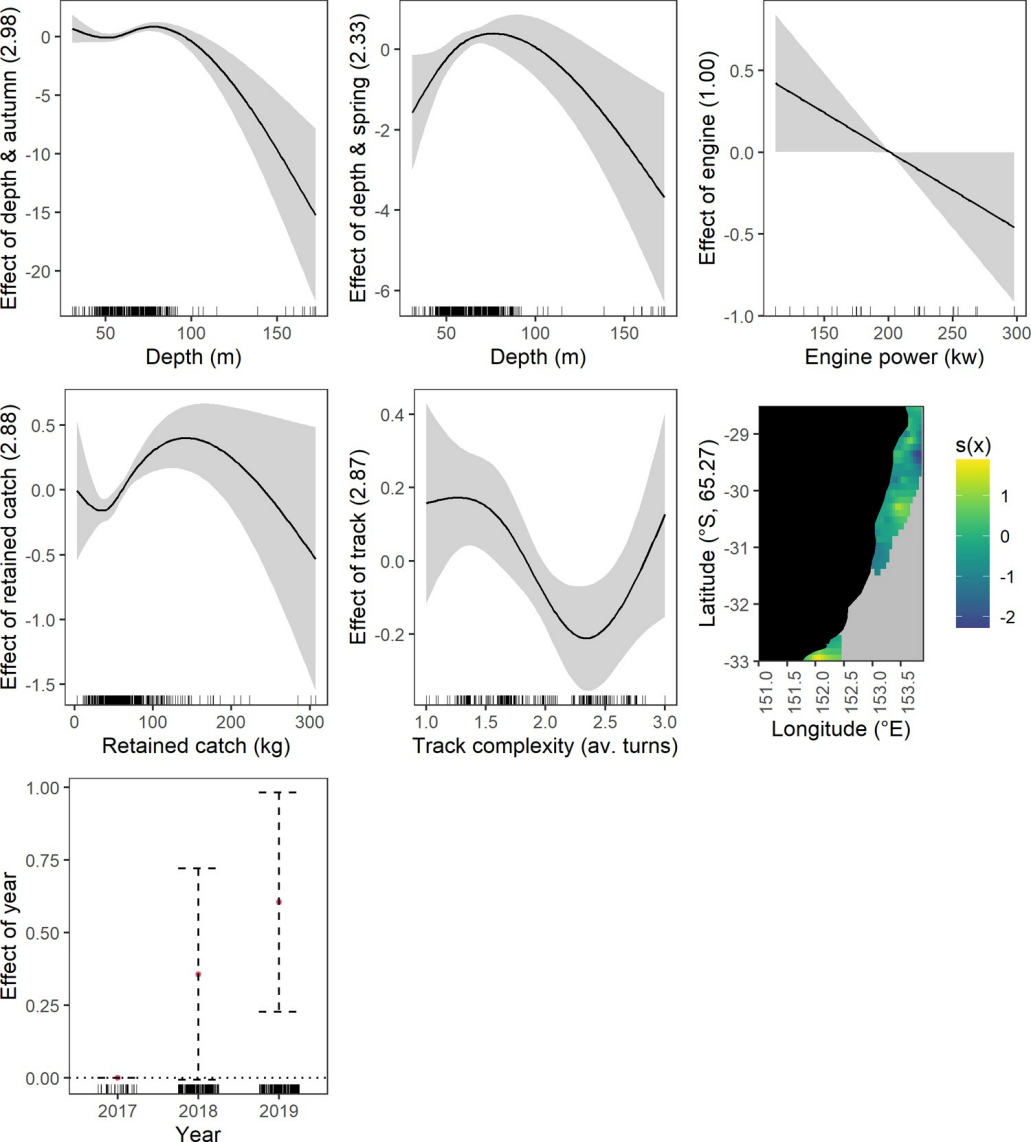
Significant effects plots from the discarded commercial crustacean response generalised additive mixed models, shaded area represents the 95% confidence limits whilst the surfaces represent the effect of 2-d smoothing on spatial coordinates.

**Fig 6:**
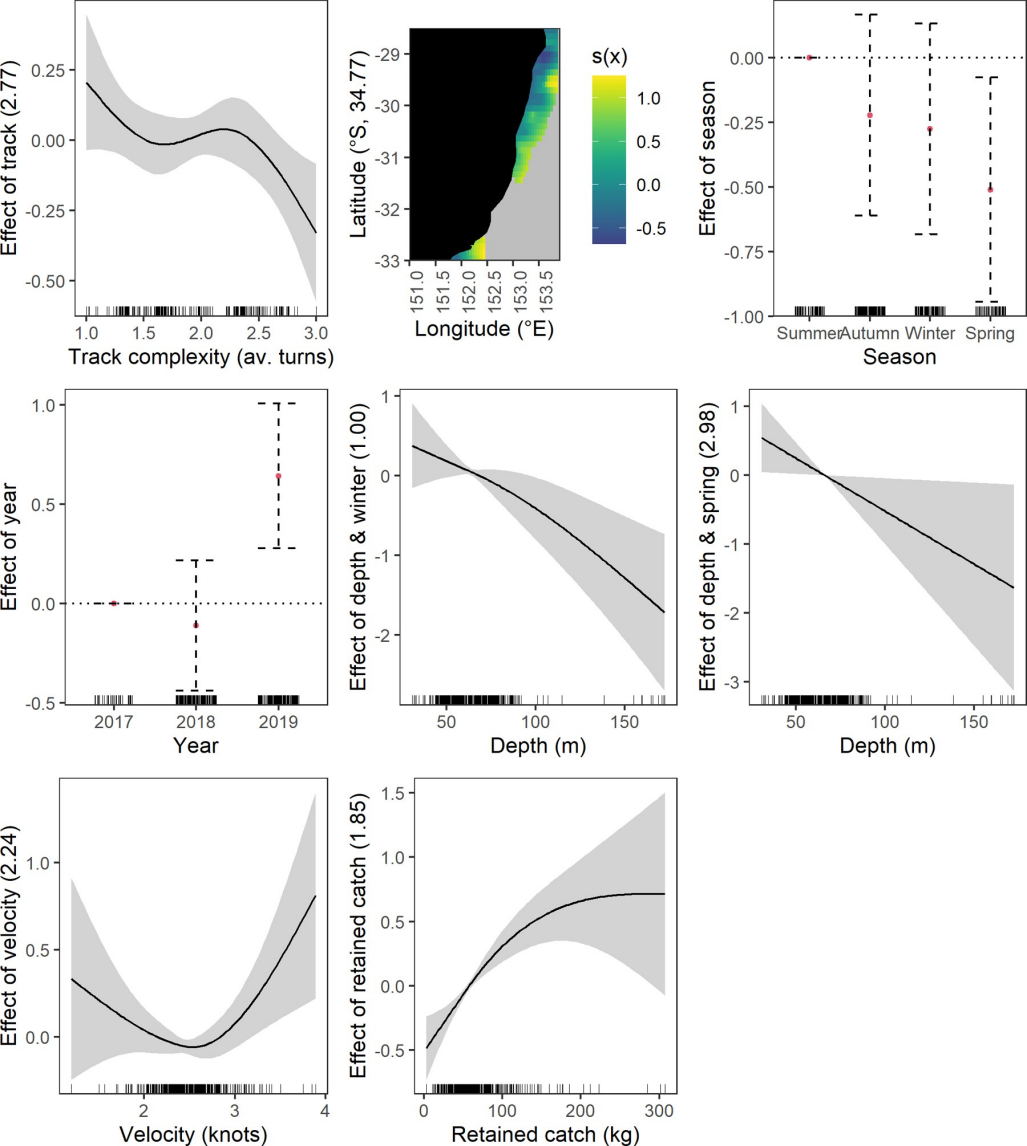
Significant effects plots from the discarded commercial fish response generalised additive mixed models, shaded area represents the 95% confidence limits whilst the surfaces represent the effect of 2-d smoothing on spatial coordinates.

*Spatial—latitude and longitude*. The spatial effect on discarding varied among species groups (Figs 2–6). Elasmobranch discarding was consistent along the entire north to south extent of the OPT with discarding greatest inshore at 30.0°S (± 1°S) (Fig 2). Crustacean discarding was also along the north to south extent but not in an area from ~ 29.5 to 30.0°S (Fig 3). Again, discarding was generally greatest inshore but lacked the spatial consistency of elasmobranch discarding. Fish discarding was slightly higher in the north (29.0 to 30.0°S) of the OPT (Fig 4), there was no effect of longitude on fish discarding. Commercial crustaceans were discarded in three small areas (Fig 5). This was strongest in the southernmost part of the fishery and offshore of ~ 30.5°S. Commercial fish were also discarded in multiple areas including offshore of 29.5°S with a narrow ridge extending southward on the offshore extremity of trawling to ~ 31.5°S. In the far south of the OPT (the southern zone), commercial fish discarding was very strong between 32.5°S and the southern-most extent of the OPT, except for a relatively small area at ~ 33.0°S and between 151.75 and 152.0°E where there was no discarding (Fig 6).

*Temporal–year*, *season and season and depth interaction*. More elasmobranchs were discarded in 2018 and 2019 compared to 2017 (Fig 2). Also, more were discarded in winter and spring compared to summer. Fewer crustaceans were discarded in autumn and spring compared to summer (Fig 3). There was a positive relationship between crustacean discarding and increasing depth during: summer across the depth range, autumn between ~ 50 to 100 m, winter at depths below 100 m and spring at depths below 70 m. There was greatest uncertainty in crustacean discarding at the deeper extremities evidenced by the wider confidence intervals (Fig 3).

Fewer fish were discarded in all seasons compared to summer (Fig 4). A positive relationship between discarding and depth occurred in summer and autumn with the relationship linear in autumn and with a sharp increase in slope at ~ 100 m deep in summer. The discarding and depth relationship was reversed in winter and spring where a negative linear relationship was evident at depths > 70 m (Fig 4). There was no clear relationship at depths < 70 m for winter and spring.

Commercial crustaceans were discarded more in 2018 and 2019 compared to 2017 (Fig 5). There was a negative discarding and depth relationship in autumn and spring. Fewer commercial fish were discarded in spring compared to summer (Fig 6). There was a negative discarding and depth relationship during winter and spring, however, again there was no clear relationship at < 70 m in winter. The greatest uncertainty was again at deeper depths (Fig 6).

#### Operational driver effects

*Track complexity*. Increasing track complexity generally had a negative relationship with discarding of elasmobranchs and commercial taxa (crustacean and fish) (Figs 2, 5 and 6). A near linear relationship was found for elasmobranchs and commercial fish, however, the spline shows a brief plateau between 1.5 and 2.5 mean turns for commercial fish (Fig 6). Discarding of commercial crustaceans had a negative relationship with increasing track complexity but at 2.5 mean turns the relationship changes to positive (Fig 5).

*Vessel engine capacity and trawl speed*. The only effect of engine capacity on discarding was for commercial crustaceans (Fig 5). Increasing capacity had a negative linear relationship with discarding. Trawl speed only affected commercial fish discarding (Fig 6). The effect was non-linear with an initial negative relationship to mid speed ranges and then positive.

*Retained catch weight*. Discarding of all species groups except elasmobranchs generally had a positive relationship with increasing mean retained catch weight (Figs 2–6). Crustaceans had a near linear relationship except at higher retained catches (Fig 3). Fish also had a near linear relationship (Fig 4). Commercial crustaceans had a near linear positive relationship with discarding to 50 to 150 kg thereon the relationship changed to negative but with increasing uncertainty (e.g. widening of confidence intervals) (Fig 5). Discarding of commercial fish had a near linear positive relationship with retained catch but plateauing after ~ 200 kg (Fig 6).

### Hypothesis 2

#### Exploratory analysis of fish and all species groups (combined) discarding and retained catch

**Fig 7:**
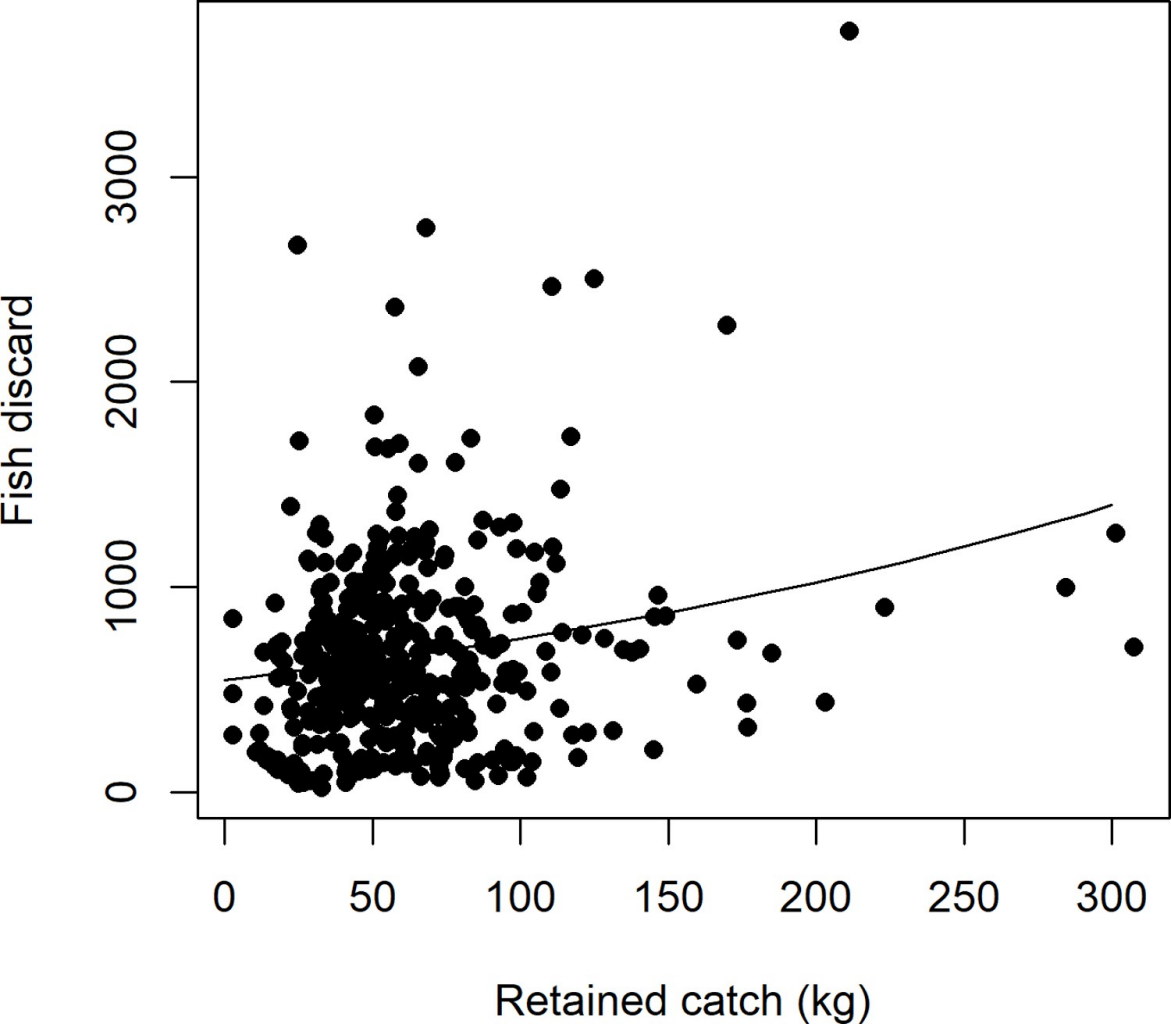
Relationship between log standardised count of discarded fish and net haul level weighted mean of retained catch.

**Fig 8:**
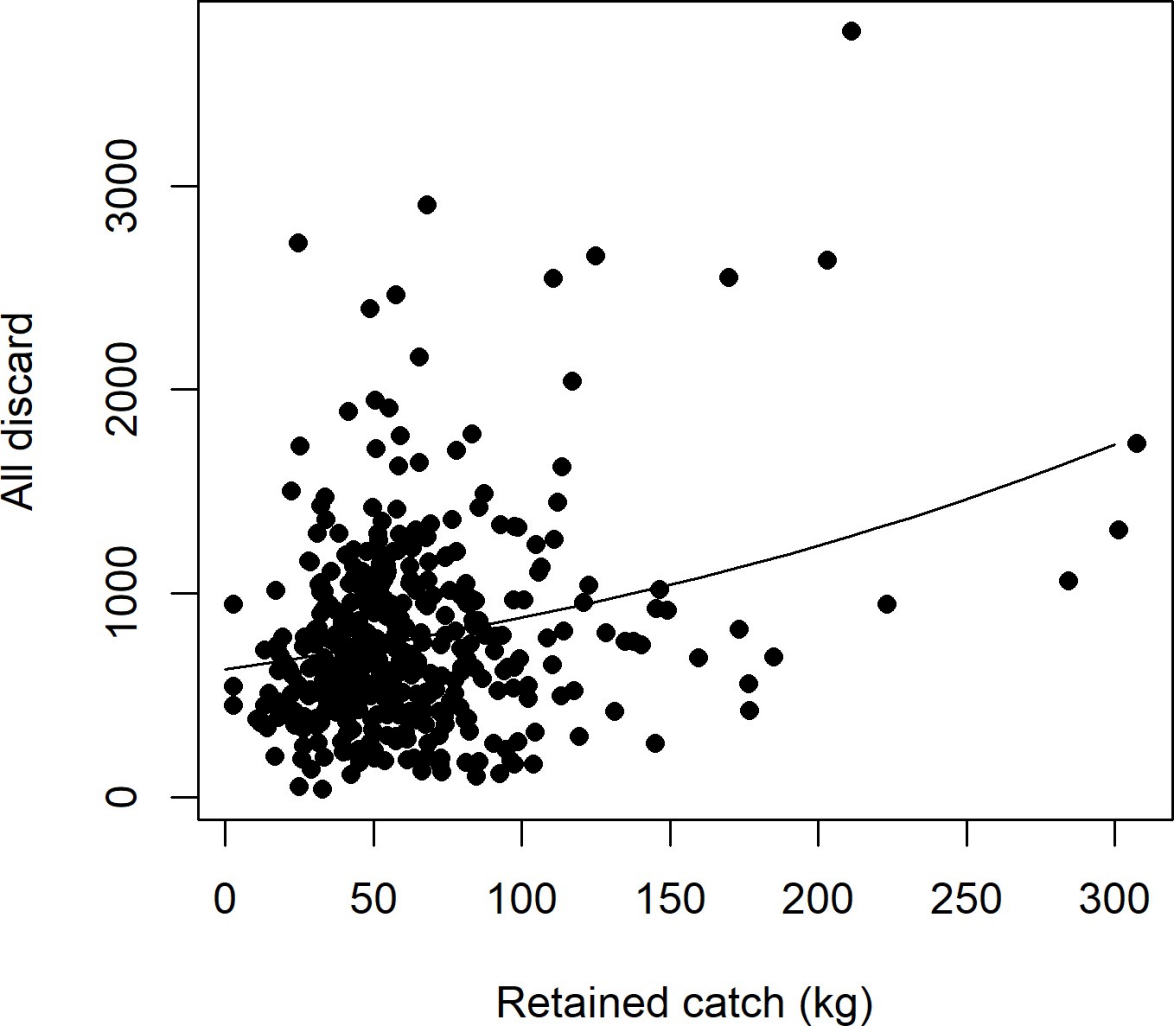
Relationship between log standardised count of all discarded functional groups (fish, crustaceans and elasmobranchs) and net haul level weighted mean of retained catch.

The fish and combined species groups discarding had a significant positive linear relationship with the mean weight of retained catch (Figs 7 and 8). Therefore, the null hypothesis of no relationship is rejected. However, the relationship was relatively weak for both fish and all species groups combined (r^2^ 28 and 35% respectively). Residuals were checked for model performance in the same manner as hypothesis 1 and no concerning structure was found (see S3 Fig).

## Discussion

### General

The present study identified environmental and operational drivers of discarding in the OPT. These drivers provide possible avenues for mitigation to reduce the potentially harmful practice of discarding. A positive relationship between the retained and discarded catch was identified but was relatively weak and may not represent a simple function of these variables. This result suggests that discard estimates based solely on the retained catch would be inaccurate for the OPT and possibly other similar demersal trawl fisheries. Our results provide a case study for other penaeid and demersal trawl fisheries.

### Hypothesis 1

The null hypothesis of no relationship between environmental and operational drivers and discarding was rejected. Environmental and operational drivers caused significant variation in discarding. However, the combination of drivers and the types of relationships varied among the five species groups. The present study is the first to model drivers of discarding in the OPT in 25 years [see 38]. The lack of model based studies on the OPT and globally (Table 4) is concerning, given the relatively high rate of discarding reported recently [e.g. 10] and it’s negative impact (e.g. waste of resources, loss of social license for trawl fisheries, impact on the ecosystem). Most previous studies investigated environmental drivers with only a few including operational drivers (Table 4 but see [6]). [6] Investigated both environmental and operational drivers and reported significant relationships between both types of drivers and discarding.

**Table 4:** Sample of studies that model the rate of discarding in demersal trawl fisheries.

Model	Environmental factors	Operational factors	Gear factors	Other factors	References
ANOVA	**Location, season, year**				[38]
ANOVA	Area, season			**Year class strength**	[49]
GLM	**Depth,** location	Net position	**BRD, TEDs**		[50]
GAMM	**Location**, **year**, **depth**, season	**Total catch**, **engine capacity**		**Juvenile abundance, quota utilisation**	[6]
GAM	**Depth, season**				[51]
GLMM	Depth (41–68 m)	**Headline length**, **tow duration**, speed*, **total catch**	**BRD** (square mesh)		[52]
GLM	Location	Net position	**BRD**, TEDs*		[42]
GAM^E^ & regression^O^	**Year, season, depth, location**	Various vessel^V^, **landings value**			[35]
GAM and spatial analysis	Chl-a, **SST, depth, location, season**			Effort	[53]
GAM	**Salinity**, **TSS**, **temperature**, Chl-a, season, **year**				[54]
GLMM	**Lunar**		**BRD**	Sector	[34]

Only main effects are described (i.e. no interactions or random effects) and Chl-a denotes productivity. Bolded indicates significant factor. Model acronyms are analysis of variance (ANOVA), generalised linear model or mixed model (GLM and GLMM respectively) and generalised additive model or mixed model (GAM and GAMM respectively). ^V^ vessel capacity, vessel energy consumption and vessel annual landing. ^E^ denotes environmental model and ^O^ operational. BRD is bycatch reduction device.

#### Environmental drivers

The trawling location on the NSW north coast significantly affected the numbers of organisms discarded for all species groups tested. Spatial variation in discarding has been reported previously in the OPT [see 38] and similarly by research on other demersal trawl fisheries (e.g. [6], Table 4). The relationship between the discarding of fish and latitude is simple, partly due to the lack of interaction with longitude, and shows a north to south negative gradient. It is feasible that the relationship could be underpinned by the increase in fish species richness and abundance in a northerly direction due to the increase in water temperature and habitat diversity [55,56]. The finding is supported by the northern zone in the present study recording 33 more species than the southern zone (see Table 2) and the East Coast Prawn Trawl fishery (QLD EKP fishery component) discarding more fish on a lower latitude section of the same coastline [10]. Latitude and longitude interacted to affect the discarding of all other species groups. The location effect was mainly different but there was a rough pattern of more consistent discarding of elasmobranchs and crustaceans whereas the commercial species groups were discarded in areas (herein hotspots). Elasmobranch diversity is generally greatest nearshore as diversity is bolstered by offshore species using nearshore areas as nurseries [57]. Elasmobranchs and some commercial species are under pressure from anthropogenic activities globally [58], understanding the location effect on discarding could aid their population persistence. Unfortunately, elasmobranchs weren’t analysed statistically in the previous OPT discard modelling project [see 38], therefore, changes in the effect of location on elasmobranch discarding over time cannot be elucidated. However, [38] and another study (a small-scale investigation into the effects of subsampling of bycatch) that included trawling in the OPT extent reported that elasmobranch captures were substantial (~ 20% of total [59]). As mentioned above, commercial species (crustaceans and fish) were discarded in location hotspots, a location effect was also reported by [38]. [38] show a negative north to south gradient of commercial species discarding based on observer sampling out of four ports separated by latitude. The north to south commercial species discarding gradient isn’t represented by the spatial analysis in the present study, although the species richness reported here does follow the latitudinal gradient (Table 2). The lack of similarity in the spatial effect of discarding of commercial species could be for at least two reasons. First, marketability of species has changed over the last few decades with many more species now in demand which has changed discarded bycatch communities. Second, there have been numerous MPAs implemented between studies (e.g. Solitary Islands Marine Park) meaning some grounds that were fished in [38] were not in the present study and the punctuation of trawling grounds by MPAs has created the hotspots. The effect of discarding commercial species is complex because both fisheries and the underlying marine ecosystem could be negatively affected by such discarding [9]. As such, more research is warranted to determine the impact of the OPT on elasmobranchs and commercial species and is discussed in more detail below (see Optimising Mitigation and Future Research).

Time drivers, (year and season), including a season depth interaction, were found to significantly affect the discarding of certain species groups. They have been found to influence discarding in other model-based studies [see 56] (Table 4). The effect of year on decreasing commercial fish discarding coincides with falling EKP catches [60]. Fish were more likely to be discarded in summer, likely due to warm currents in the OPT extent facilitating relatively rich and abundant fish fauna [38]. Previous research has described an increase in assemblages and abundance of fish in coastal NSW in summer due to the timing of recruitment [61] and overlap of tropical and temperate species [62]. Although, another important consideration is market forces (and other socioeconomic factors) on the fisher’s decision making with a large summer coastal population in NSW increasing demand for seafood and EKPs fetching a premium price at times of high local demand such as Christmas. Approximately, 130 tonnes of NSW prawns were to be sold between 21–23 of December 2019 making prawns the third most popular Christmas seafood item [63]. Like space, time and its interactions, could provide mitigation options (e.g. dynamic closures, [see 64]) and may aid in addressing complexities in spatial closures effectiveness across species groups. The success of this type of intervention will depend on relative weights of environmental and socioeconomic drivers.

#### Operational drivers

A positive retained catch weight and discarding relationship was identified by the present study for all species groups, except elasmobranchs. A relationship of this nature has been described multiple times in the literature [6,56,65] (Table 4). There are some plausible theories describing why there might be such a relationship. Large catches may cause the gear to reduce selectivity either by blocking of the codend or a mechanical reduction of the mesh when the gear is under increased load [56,66]. However, the loss of selectivity would more likely be a logarithmic relationship [56] (rather than the observed linearity), although, it is possible that there is a counter force from increased net drag with increasing load causing a reduction in spread and therefore net sweep. Also, trawls with BRDs such as the square mesh panel in the OPT are unlikely to become blocked, at least by smaller bodied organisms. More of the catch, particularly fragile species such as some small commercial fish (e.g. trawl whiting, *Sillago robusta* and *Sillago flindersi*), can become damaged in a heavily laden net and thus lead to increased discarding, also, larger catches may increase economic discarding to avoid oversupply [56]. Further, small vessels are limited in what they can hold [67] and intuition suggests large catches may mean a generally more productive fishing ground. The range of theories suggests it is hard to determine an exact cause, a fishery independent research program could provide some answers, by removing the socioeconomic component [65]. It is possible that large amounts of rays and other dorso-ventrally flattened species could block the net (despite square mesh BRDs) and crush the commercial taxa, leading to increased discarding, therefore avoiding areas where rays are abundant could be worthwhile. However, our data suggests a weak relationship at best (S4 Fig) although blocking of the net may be caused by a range of ventrally flat organisms (e.g. sponges that are not reflected in our data). The inclusion of TEDs has been shown to assist in reducing the capture of elasmobranchs [68] and could play a role in mitigating these issues (see Optimising Mitigation below). The retained weight driver of discarding is perhaps limited as a mitigation tool due to the variety of potential causes.

Operational drivers track complexity, trawl speed and engine capacity mainly influenced the discarding of commercial species groups. These type of drivers have received little attention in the literature but [52] tested the effect of trawl speed on discarding and others have expressed the research need [e.g. 1]. Increasing track complexity, speed and capacity generally had a negative relationship with discarding for the relevant species groups. The negative relationship with track is the opposite to what was expected. The expected result was that increasing track complexity and speed would increase discarding due to greater interactions with organisms. For example, our hypothesis was increasing track indicates the presence of structure (hence complex habitat) and a faster moving net would capture more but our discard models do not substantially support this hypothesis. However, commercial fish discarding was positively influenced by trawl speed from 3 to 4 kts. A greater number of fish, including below MLS fish, are likely captured in faster moving gear as it becomes increasingly difficult to avoid [69]. Increasing trawl speed has been shown to increase the width of the trawl net in penaeid [47] and other demersal trawl fisheries [e.g. 70] but any effect on discarding is not clear in the present study. Faster and larger trawls sometimes catch pelagic sharks [68]. Only, six pelagic sharks were caught in the present study (e.g. 3 thresher sharks, *Alopias vulpinus*) with all trawl velocities around the mean (~ 2.4 kts) or higher. As mentioned, discarding of commercial crustaceans and commercial fish is particularly problematic. The present study recorded the discarding of a variety of commercial (and recreational) finfish such as teraglin (*Atractoscion aequidens*), snapper (*Chrysophrys auratus*), and flathead (*Platycephalus* species) (etc.). The fishery status of these species is classed as sustainable in NSW [71–73]. However, to help ensure the continued sustainable exploitation of these fish, research and subsequent mitigation should be a priority (see Optimising Mitigation below), especially given fishing effort could change and the potential for influence of other stressors (e.g. climate change).

Engine capacity has a positive linear relationship with vessel size (S5 Fig) and larger boats have been shown to engage in less discarding in some fisheries [6,65]. The function of storage capacity leading to less discarding [6] wasn’t normally the case in the present study (i.e. only commercial crustaceans showed a relationship) and is suggestive of multiple variables driving discarding. Larger more powerful vessels have been successful in trawling a broader range of grounds in other demersal trawl fisheries [74] and in a variety of weather conditions meaning discarding likely varies between trips. Track, speed, and capacity do not provide obvious mitigation options because some momentum and turns are required for the net to function, however, in the OPT vessels had a maximum of 400 kW engine capacity until 2018 when this restriction was removed.

### Hypothesis 2

The null hypothesis of no relationship between numbers of fish and all species groups discarded and weight of total catch was rejected; however, the relationship was relatively weak. Near linear positive relationships of catch weight on discarding of fish has been reported previously (see [6]). Further evidence is provided by the fish discarding model from hypothesis 1 which did display a positive linear relationship with mean retained catch weight. However, this relationship considers the other drivers (i.e. significant environmental factors). The similarities between the model results (see Figs 7 and 8) highlight the substantial contribution of fish to the overall volume of discarding. The theories on the relationship, such as large catches leading to more discarding due to changes in net functionality and damage to sensitive taxa due to a large volume in the net (see hypothesis 1 above), suggest that this is not a simple function. However, the discard to retained catch ratio is the most commonly used statistic to determine fishery wide discarding from logbook data in unmonitored fisheries [10,34]. Whilst it initially appears this extrapolation would be reasonable in the OPT, our further inspection of the relationship, suggests it is not a simple scale up to the fishery wide level and may cause incorrect decision making. The lack of a strong relationship highlights the need to test each fishery, preferably, with observer (or other high quality) discard data collection programs.

### Discarding in the OPT over 25 years

A substantial temporal period has passed between the present study and the previous large-scale observer-based investigations into discarding in the OPT (~ 25 years, [38]). During this period effort has reduced by ~ 20 000 trawl trips per year. The effort reduction is for a variety of reasons but mainly due to a lack of vessel replacement in an ageing fleet. Unfortunately, there are some differences limiting direct comparisons in discarding between the two studies. For example, the previous work didn’t record the number of non-fishery species, elasmobranchs weren’t included in modelling (etc.). A basic 25 year comparison based on catch to bycatch ratios suggests the contemporary OPT has a similar discarding footprint. For example, discarded to retained catch ratios are very similar, with 1.16–5.14:1 (depending on the base ports of vessels) reported in [38] and 2.1:1 in the present study whilst less overall bycatch to EKP is now the case (10.4:1 in [38] to 5.8:1 present study). The near 50% reduction in overall bycatch is likely the result of BRDs (e.g. legislated square mesh panel) introduced during the 25 year lead time [9,52]. Suggesting that the northern NSW marine ecosystem which interacts with the OPT is being maintained. The effort reduction in the OPT should have played a role in the maintenance of the ecosystem but may have been offset slightly by the concurrent increase in fishing power (e.g. improvements in positioning systems). Clearly there is more work to be done to better understand the impact of trawling and particularly discarding on the marine ecosystem in northern NSW.

### Optimising mitigation

As drivers of discarding were identified for multiple species groups then ideally mitigation methods would reduce discarding of all groups. However, drivers often differed among species groups or the relationship between drivers and discarding differed at the species group level. Therefore, identifying a global driver and associated mitigation method is ambitious. A combined approach is more likely to reduce discarding of multiple species groups, for example, combining dynamic closures, quotas (species and effort), and BRDs could overcome the limitations of anyone approach. Some species quotas were introduced in 2019 to the OPT, these were aimed at increasing profitability but may have a secondary benefit of reducing discarding. Another approach to mitigating discarding may be to prioritise certain species groups. For example, commercial crustaceans, commercial fish, and elasmobranchs could be targeted in discarding reduction intervention programs. Our modelling suggests reduced discarding of these species groups would occur if trawl tows were done in depths > 65 m and at velocities < 3 knots. This concept requires further research to address feasibility. For example, how would this affect profitability? As more fuel would very likely be consumed trawling on deeper grounds. Other studies from the QLD section of this fishery identified shallow depths as discarding hotspots [42,50] suggesting this driver is worth further investigation. Queensland studies were in more tropical low latitudes than the present study and may account for our reporting of a variety of discarding relationships (i.e. species group dependent interactions due to greater effect of seasonality). Perhaps some shallow water discarding hotspots in the OPT could be targeted for discarding intervention, our results indicate that there were significant seasonal and shallow water hotspots of fishery species discarding. However, closures can cause economic hardships for fishers [9]. Spatial interactions with season and depth suggests closures could be dynamic and therefore help alleviate economic hardship. Some dynamic spatial closures are already operational in the OPT to protect single species [64]. It is timely to find mitigation methods that reduce discarding of a greater proportion of species groups. The global issue of elasmobranch decline suggests that implementing TEDs to mitigate discarding in the OPT warrants investigation. Currently TEDs are not legislated for deployment in the OPT due to low interactions with turtles [36]. Like the other mitigation concepts mentioned, TED deployment would require more research, especially as BRDs have been found to have some inherent problems such as a negative effect on trophic structure [75] and devolving benefits sometimes due to fisher modifications [31].

Some fisheries have selected single mitigation approaches which have not had the desired result. For example, the proactive EU approach to discarding management (discard ban called Landing Obligation (LO)) saw the transition where Total Allowable Catches went from landings only to total catches [76]. The LO began implementation in 2015 and is now fully operational. To date, the LO has not had the desired effect of reducing discarding [see 76] mainly due to noncompliance and exemptions. Research has suggested other discard mitigation methods would be more likely to have the desired result [35]. A combination of remote electronic monitoring (REM) and LO could increase efficacy as it may alleviate compliance issues [see 77].

Issues with previous mitigation attempts (e.g. BRDs, LOs etc.) highlight the complexities involved with reducing discarding. In the OPT effort reduction has likely set the mitigation of discarding on the correct trajectory [1,51] with further reductions likely from targeting the drivers identified by the present study. However, to realise a reduction of discarding based on a combined mitigation approach will require investment (further research etc.). Broadly, it is likely that the best mitigation method to reduce discarding will be fishery specific. For the OPT, more frequent timeseries analysis of discarding will aid in targeting mitigation and determine the urgency of intervention.

### Future research

The present study, combined with other similar research [e.g. 6] demonstrates that discarding in demersal trawl fisheries is complex (i.e. multiple drivers of discarding). Therefore, more research is required to provide mitigation methods to ensure the sustainability of marine ecosystems interacting with trawl fisheries. Additionally, research and monitoring programs will also ensure the accurate quantification of the volume of discarding in the future. The mixed messages on the effectiveness of mitigation measures and the likely species specific (or at least species group) efficacy highlights the requirement for regular monitoring. Especially given that the onset of climate change is gathering momentum globally and the environment along the OPT extent is one of the most rapidly changing [78]. Twenty-five years between monitoring is too long considering the speed of climate driven (and other) change. For the OPT, observer monitoring should take place every five years or deployment of REM is potentially another option. Thus, building a timeseries of discarding data to facilitate timely intervention where concerning discarding trends are discovered and, also, reducing issues such as devolving BRDs (etc.). Modern monitoring approaches such as REM could help alleviate the strain on resources caused by regular monitoring. Demersal trawling may also interact with benthic habitat which could have a negative impact on the marine ecosystem and has never been investigated in the OPT. Further, some of the discarding complexities could be removed by implementing a fishery independent monitoring program (i.e. remove the socioeconomic component) and multivariate analysis (i.e. identify species that influence drivers of discarding). Thus, benefiting the deployment of drivers in mitigation. Other research to understand discarding is the effect of recent species quotas, effort reduction versus increased fishing power, and impacts of BRDs on trophic structure. Ensuring the robustness and repeatability of data between monitoring programs to ensure comparability should also be a priority.

### Conclusion

The global problem of fishery discarding continues despite developments in the last 20 years. Therefore, it is timely that research like the present study gathers momentum. Regular monitoring and research are required to continue the development of mitigation methods in the OPT and other demersal trawl fisheries. Not just to inform a reduction of discarding program but to monitor the amount of discarding over time. The OPT discarding and associated ecosystem appears at least stable (i.e. via our simple temporal comparison), bolstered by effort reduction and (more recently) potentially by species quotas. However, collection of timeseries data to better assess the health of the interacting ecosystem should be prioritised to guide decision making (i.e. how to mitigate and when). The present study has provided new leads (i.e. identified space, time and depth discarding hotspots and operational drivers) for mitigating discard in the OPT and other similar fisheries. Possibly, combining a suite of mitigating measures is the best way to reduce discarding in the OPT but this hypothesis and other fishery performance metrics would be greatly informed by high quality timeseries information.

## Supporting information

S1 Fig Mean (± SD) depth trawled for each observed trip (three trawls per trip), for clarity panels show a depth range with a mean depth of < = 50, > 50 to < = 60, > 60 to < = 80 and > 80 m from top to bottom. (JPG)

S2 Fig Relationship of residuals and linear factors for fish generalised additive mixed models (hypothesis 1) as an example (representative) of model assessment. (JPG)

S3 Fig Relationship of residuals and linear factors for fish counts and mean retained catch weight generalised linear mixed models (hypothesis 2) for model assessment. (TIFF)

S4 Fig The effect of elasmobranch discarding on total discarding of all organisms with loess smooth. (TIFF)

S5 Fig Relationship (with loess smooth) between trawl vessel length and engine capacity for vessels participating in the ocean prawn trawl observer study (present study). (TIFF)
