# Supplementary figures and images for: Understanding discarding in trawl fisheries: A model based demersal case study with implications for mitigating and assessing impacts
Source: PLoS One. 2022 Feb 17;17(2):e0264055. doi: 10.1371/journal.pone.0264055 (PMC8853496; doi:10.1371/journal.pone.0264055)

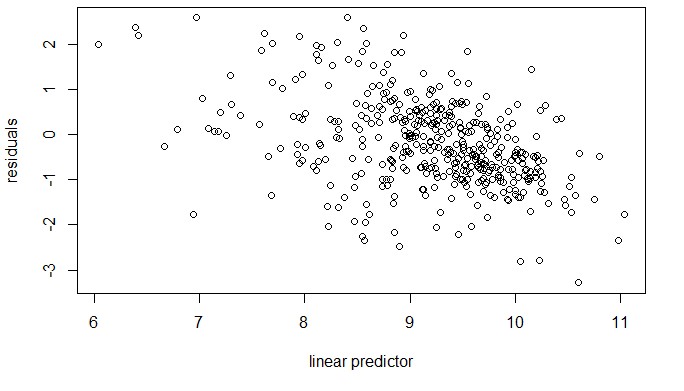

Supplement: S1 Fig — (JPG) [file pone.0264055.s001.jpg]

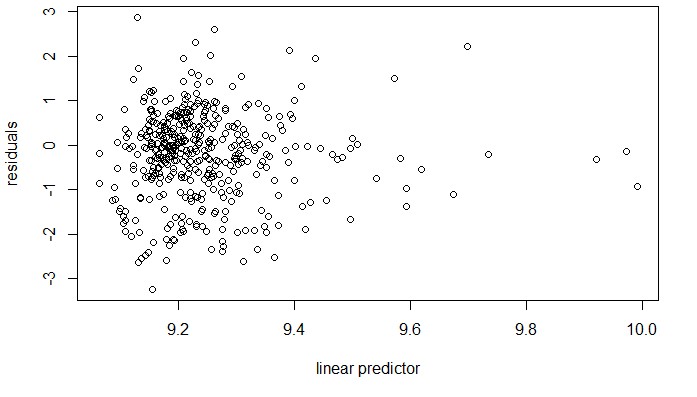

Supplement: S2 Fig — (JPG) [file pone.0264055.s002.jpg]

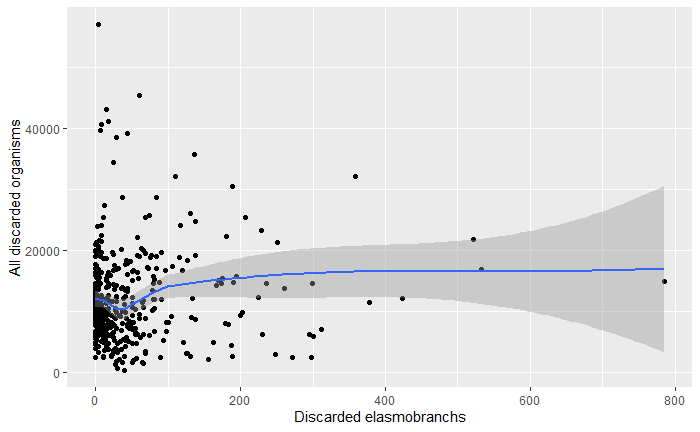

Supplement: S3 Fig — (TIFF) [file pone.0264055.s003.tiff]

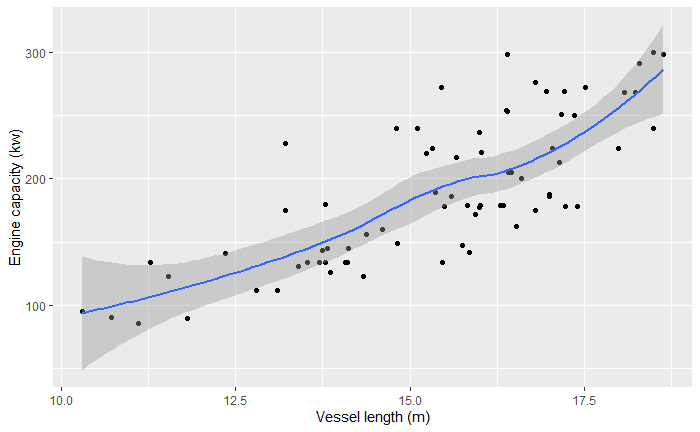

Supplement: S4 Fig — (TIFF) [file pone.0264055.s004.tiff]

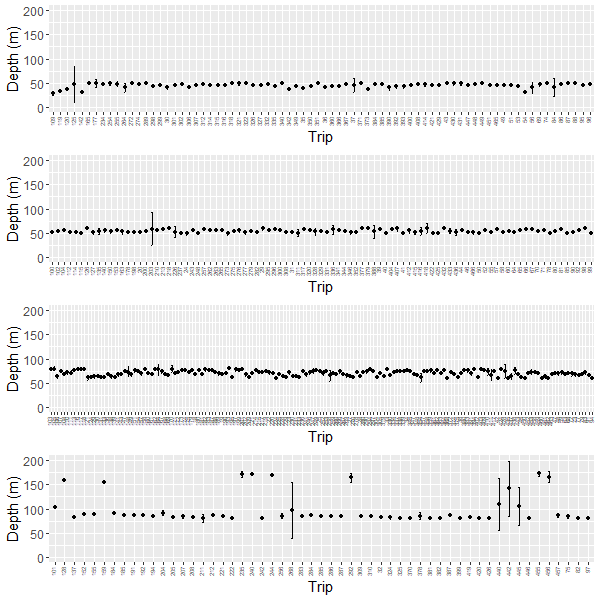

Supplement: S5 Fig — (TIFF) [file pone.0264055.s005.tiff]
